# Neural mechanisms of expert persuasion on willingness to pay for sugar

**DOI:** 10.3389/fnbeh.2023.1147140

**Published:** 2023-03-13

**Authors:** Ioannis Ntoumanis, Alina Davydova, Julia Sheronova, Ksenia Panidi, Vladimir Kosonogov, Anna N. Shestakova, Iiro P. Jääskeläinen, Vasily Klucharev

**Affiliations:** ^1^International Laboratory of Social Neurobiology, Institute for Cognitive Neuroscience, HSE University, Moscow, Russia; ^2^Brain and Mind Laboratory, Department of Neuroscience and Biomedical Engineering, Aalto University School of Science, Espoo, Finland

**Keywords:** expert persuasion, sugar, EEG, healthy eating, machine learning, intersubject correlation, willingness to pay, social influence

## Abstract

**Introduction:** Sugar consumption is associated with many negative health consequences. It is, therefore, important to understand what can effectively influence individuals to consume less sugar. We recently showed that a healthy eating call by a health expert can significantly decrease the willingness to pay (WTP) for sugar-containing food. Here, we investigate which aspects of neural responses to the same healthy eating call can predict the efficacy of expert persuasion.

**Methods:** Forty-five healthy participants performed two blocks of a bidding task, in which they had to bid on sugar-containing, sugar-free and non-edible products, while their electroencephalography (EEG) was recorded. In between the two blocks, they listened to a healthy eating call by a nutritionist emphasizing the risks of sugar consumption.

**Results:** We found that after listening to the healthy eating call, participants significantly decreased their WTP for sugar-containing products. Moreover, a higher intersubject correlation of EEG (a measure of engagement) during listening to the healthy eating call resulted in a larger decrease in WTP for sugar-containing food. Whether or not a participant’s valuation of a product was highly influenced by the healthy eating call could also be predicted by spatiotemporal patterns of EEG responses to the healthy eating call, using a machine learning classification model. Finally, the healthy eating call increased the amplitude of the P300 component of the visual event-related potential in response to sugar-containing food.

**Disussion:** Overall, our results shed light on the neural basis of expert persuasion and demonstrate that EEG is a powerful tool to design and assess health-related advertisements before they are released to the public.

## 1. Introduction

The obesogenic environment in which consumers make food choices makes it difficult for them to maintain their healthy eating goals (de Ridder et al., 2017). Public health measures have failed to provide such support, since obesity rates are rising rapidly with far-reaching health consequences (Kelly et al., 2008; Dixon, 2010). Although sugar is a key cause of obesity (Yu et al., 2022), there is limited research exploring what can influence individuals to consume less sugar. We have recently demonstrated that a healthy eating call by an expert can significantly decrease the willingness to pay (WTP) for sugar-containing food (Ntoumanis et al., 2022). Here, we expand this line of research by investigating the neural correlates of this phenomenon, that is, which aspects of neural responses to the same healthy eating call can predict the efficacy of expert persuasion.

Despite the rapid growth of understanding of what can help consumers to make healthier food choices (Higgs, 2015; Leng et al., 2017; Cadario and Chandon, 2020), little is known about how nudge interventions can affect sugar consumption. Previous studies on this topic have mainly examined such eating nudges as health-related labels and visibility enhancements, with the results being inconsistent (Mai and Hoffmann, 2015; Bialkova et al., 2016; Romagny et al., 2017; Donnelly et al., 2018; Thiene et al., 2018; Drugova et al., 2020; Potthoff et al., 2020; Schubert et al., 2021). Critically, the types of nudges mentioned above are, in general, half as effective as healthy eating calls, i.e., written or oral injunctions aiming at changing unhealthy food choices (Cadario and Chandon, 2020). Indeed, healthy eating calls have successfully been used to reduce unhealthy food choices both in laboratory-based studies (Binder et al., 2020; Ha et al., 2020) and in field experiments (Mollen et al., 2013; van Kleef et al., 2015). However, only recently this type of intervention was applied for the first time against sugar consumption in laboratory settings (Ntoumanis et al., 2022) and its effectiveness was significant. In fact, the results suggested that a healthy eating call (first-person narrative) by a health expert decreased the WTP for sugar-containing food. Here, by using the same healthy eating call in a slightly different experimental design, we hypothesized that (H1) the healthy eating call would decrease individuals’ WTP for sugar-containing products.

Recently, electroencephalography (EEG) is used more and more to predict consumers’ preferences and choices (e.g., Hakim et al., 2021; Mashrur et al., 2022; Raiesdana and Mousakhani, 2022). EEG offers an opportunity to overcome the biases inherent in self-reports, such as dishonesty (Tourangeau and Smith, 1996), while at the same time, it allows to investigate the neural mechanisms underlying consumer behavior (Lin et al., 2018). An EEG-derived measure that is being increasingly used in this research area is the similarity of individuals’ neural activity, or intersubject correlation (ISC). As a marker of engagement and attention (Hasson et al., 2004; Dmochowski et al., 2012; Ki et al., 2016), ISC has successfully been used to predict population-wide music popularity (Leeuwis et al., 2021), movies’ box-office performance (Christoforou et al., 2017) and individual preferences for television ads (Dmochowski et al., 2014). Given these findings, ISC is considered to be a promising neurophysiological measure of advertising effectiveness in social contexts (Pozharliev et al., 2017), especially when the study designs include long-duration stimuli (Hakim and Levy, 2019). Here, we used EEG ISC to predict the efficacy of expert persuasion reflected in the change of the WTP following the intervention. In fact, we hypothesized that (H2) high ISC during listening to the healthy eating call would result in a large decrease in the WTP for sugar-containing products.

In addition to the similarity of neural responses to the healthy eating call, we also examined whether spatiotemporal patterns of EEG signals are predictive of expert persuasion, *via* a multivariate pattern analysis (MVPA). MVPA is typically used to decode the difference between conditions or groups of subjects, based on the observed spatiotemporal patterns of brain responses (Fahrenfort et al., 2018). Therefore, it allows quantification of experimental effects without *a priori* electrode selection. Previous EEG studies have used this methodology to successfully predict subsequent ratings of stimulus attributes (Bode et al., 2014; Turner et al., 2017), as well as decision-making related to the stimulus (Bode et al., 2012; Charles et al., 2014). Despite the increasing popularity of MVPA in EEG event-related studies (e.g., Turner et al., 2017) and fMRI studies using naturalistic stimuli (e.g., Saarimäki et al., 2016), it has not yet been applied to EEG studies using naturalistic stimuli. Given the multivariate nature of EEG (Peters et al., 1998), we consider this an important gap in the field.

Thus, the goals of conducting an MVPA were two-fold. First, we aimed to demonstrate a classification pipeline as a proof-of-concept for studying the EEG activity underlying the consumers’ acceptance of persuasion. Second, given that previous studies have successfully applied MVPA of EEG signals to predict decision-making related to non-naturalistic stimuli (e.g., Turner et al., 2017), we tested whether (H3) MVPA of EEG signals can also predict decision-making related to naturalistic stimuli. To reach these goals, we trained a machine learning classification model to predict the decrease in the WTP for sugar-containing products from patterns of EEG responses to the healthy eating call. Such a multivariate pattern analysis (MVPA) is usually performed in distinct time windows (Bode et al., 2014; King and Dehaene, 2014; Turner et al., 2017). If there is a statistically significant association between the variable of interest and the EEG patterns in a particular time window, then this time window is considered to contain the respective information of interest (Turner et al., 2017).

In order to better understand the neural mechanisms underlying expert persuasion, we also investigated how the healthy eating call affects particular event-related potentials (ERPs) elicited by viewing sugar-containing products that may prompt WTP decisions. ERP analysis has widely been used to discern the neural correlates of consumer behavior (for a review, see Lin et al., 2018). Of particular interest are long-lasting positive waves, such as the P300 component, which is known to reflect the allocation and maintenance of attentional resources (Polich, 2007) and was repeatedly recorded during product evaluation (Ryu et al., 2010; Wang and Han, 2014; Cai et al., 2021). Previous studies have disclosed that the P300 amplitude is increased, when seeing products that fit one’s preferences (Wang and Han, 2014), products that are recommended by others (Guo et al., 2016), products one craves (Svaldi et al., 2015; Biehl et al., 2020), and products that one is willing to buy (Jones et al., 2012; Lin et al., 2018). Since our healthy eating call aims to influence participants against sugar-containing products, we hypothesized that (H4) the P300 amplitude in response to sugar-containing food would decrease after listening to the healthy eating call. Moreover, earlier studies have speculated that P300 could predict WTP (Schaefer et al., 2016), while also being involved in social conformity (Guo et al., 2016). Therefore, we also hypothesized that (H5) the expected decrease in the WTP for sugar-containing food and the expected decrease in the P300 amplitude in response to sugar-containing food after the healthy eating call would be positively correlated with each other.

## 2. Materials and methods

### 2.1. Participants

Forty-nine participants (29 females, aged 18–40 years, mean age = 22.50) were recruited *via* online advertisements. These participants were different from those who participated in our previous work (Ntoumanis et al., 2022). All of them reported that they were right-handed, healthy, had a normal or corrected-to-normal vision, had no history of psychiatric diagnoses or eating disorders, no neurological or metabolic illnesses, and were not taking any prescribed medication. Eating sweets, in general, was also an inclusion criterion, so that we filter out potential participants who might already dislike sugar altogether. The sample size was similar to or larger than previous studies exploring the relationship between WTP for products and EEG indices (Ramsøy et al., 2018; Liao et al., 2019). Four participants were excluded from all analyses for having excessively noisy EEG data. Excluding them from both the behavioral and the EEG analyses ensured that the results were based on the same consistent sample. The final sample consisted of 45 participants (27 females, aged 18–40 years, mean age = 22.51). All participants received a flat fee of 600 monetary units (MU) equivalent to ~21.95, with the correction for purchasing power parities (OECD, 2021). Additionally, they received a reward based on their decisions in the experimental task. The mechanism of how this reward was determined was explained to them in detail in the instructions prior to the experiment.

### 2.2. Stimuli

**Figure 1:**
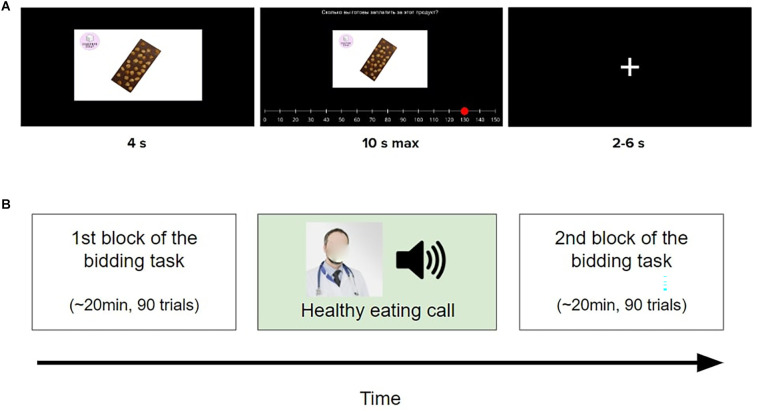
Trial structure and study design. **(A)** Sample trial of the bidding task with a product labeled as “sugar-containing”. In the beginning, the product was presented for 4 s (“early evaluation phase”). Next, a message was displayed at the top of the screen “How much are you ready to pay for this product?”. Participants had 10 s to indicate their willingness to pay (WTP) for this product. Last, a fixation cross was shown (2–6 s) and the next trial began. The trials of the other two conditions differed only in the label and in the presented product. **(B)** Experimental procedure. Participants first performed a block of the bidding task, then they listened to the healthy eating call and finally, they performed a second block of the bidding task. Here, the face of the doctor has been blurred due to copyright and ethical reasons.

Ninety full-colored photographic pictures (200 dpi) of sweets and everyday products were used. The pictures represented products without packaging (Figure 1A) to avoid any confounding effect of the package (Motoki and Suzuki, 2020). All products existed in the market during the period of data collection. One-third of the products were labeled as “sugar-free”, another third were labeled as “sugar-containing” and the remaining were labeled as “non-edible” (the latter served as the control condition). The labels were not deceptive (e.g., the products labeled as “sugar-free” were indeed sugar-free) and were presented in different colors (blue, pink, and yellow), so that the participants could better distinguish the three conditions. The colors were randomized between participants. Since the meaning of the “sugar-free” label might not be clear to all participants, we pointed out that the “sugar-free” label indicates that the product does not contain sugar, as in Ntoumanis et al. (2022). The pictures of the “sugar-containing” and the “sugar-free” products were the same as in Ntoumanis et al. (2022) and they have previously been pre-tested so that the perceived sweetness, tastefulness and healthfulness of “sugar-containing” products do not differ from that of “sugar-free” products (Ntoumanis et al., 2022).

### 2.3. Bidding task

Figure 1A illustrates the procedure in the bidding task, which was similar to that in Ntoumanis et al. (2022). At the beginning of each trial, a product was displayed for 4 s (“early evaluation stage”, Hutcherson et al., 2012). Afterward, participants had 10 s to indicate their WTP (“*How much are you ready to pay for this product?”*), in order to purchase this product at the end of the experiment (Plassmann et al., 2007; Hutcherson et al., 2012; Schmidt et al., 2018). The participants of the behavioral pilot study reported that 10 s was enough time for them to make a decision. The initial position of the marker on the WTP slider was randomized across trials (Martinez-Saito et al., 2019). The left and right keyboard keys allowed the participants to change the initial value of the slider to the value they wished, before pressing Enter key to confirm the bid. No response within the time limit resulted in a WTP of 0 MU, following previous studies (Hutcherson et al., 2012; Ntoumanis et al., 2022). The values of the slider ranged from 0 to 150 MU, with an increment of 10 MU, since this is the range of the actual prices of the products in the market. Each block contained the same amount of “sugar-free”, “sugar-containing” and “non-edible” products, with the order of the items being randomized across participants and blocks. Finally, a fixation cross was shown and the next trial began. The duration of the fixation cross was 2–6 s in order to prevent anticipation (Hutcherson et al., 2012; Schmidt et al., 2018).

At the beginning of the experiment, participants received 150 MU in cash as an endowment to use in the bidding task for purchasing products, since bidding decisions have been found to be sensitive to whether or not they are hypothetical (Lusk and Schroeder, 2006). The Becker-DeGroot-Marschak auction was employed in order to measure individual preferences and each participant’s exact WTP for every product (Becker et al., 1964; Plassmann et al., 2007). According to this auction, one trial was randomly selected at the end of the experiment. Let *b* denote the bid made by the participant in that trial. A random number *n* was also drawn from a known distribution (in our case, 0, 10,‥., 150 MU was chosen with equal probability). If *b* ≥ *n*, the participant received the product corresponding to that trial and paid a price equal to n. Otherwise, the participant did not receive the product but also did not pay anything (for a similar design, see Plassmann et al., 2007). The endowment was equal to the maximum WTP, so that participants do not have to worry about distributing their 150 MU over different products and they can treat each trial as if it were the only decision that counted (Plassmann et al., 2007; Ntoumanis et al., 2022).

### 2.4. Healthy eating call

The healthy eating call was the same as in Ntoumanis et al. (2022): an audio first-person narrative by a nutritionist emphasizing the health risks of sugar consumption. It started with an introduction of the narrator, then 13 arguments were expressed sequentially, and finally, there were some closing remarks. The arguments against sugar consumption expressed in the narrative were retrieved from scientific sources (e.g., Lenoir et al., 2007). The narrative also contained clear evidence about the nutritionist’s positive view towards sugar-free products. The narrator was introduced to the participants as a nutritionist because communicators with high expertise have been found to be particularly persuasive (Deutsch and Gerard, 1955; Binder et al., 2020; Hang et al., 2020; Ntoumanis et al., 2022). The audio version of the healthy eating call was recorded by a professional male narrator in order to maximize participants’ engagement (duration = 7 min). The healthy eating call was written and presented to the participants in their native language. The English translation can be found here: https://osf.io/894mk/. While the narrative was being played, the static image of a doctor was displayed on the screen to maximize participants’ attention (Figure 1B). The narrative we used has been shown to not induce any of the basic emotions at a considerably high level (the average rating of each emotion was lower than 2.8 on a 5-point scale, see Ntoumanis et al., 2022).

### 2.5. Questionnaires

To explore the influence of consumer heterogeneity on the efficacy of expert persuasion, participants completed four questionnaires not earlier than 2 days prior to attending the experiment. The first questionnaire assessed demographic information, including gender, age, weight, height [for the calculation of the Body Mass Index (BMI)], and level of education (four levels: incomplete secondary education, secondary education, incomplete higher education, higher education). In addition, participants completed the Conformity scale (Mehrabian and Stefl, 1995; Keller, 2019; the internal consistency in the current study, *α* = 0.747), the Consumer susceptibility to interpersonal influence scale (Bearden et al., 1989, translated by us; the internal consistency in the current study, *α* = 0.701), as well as the Big 5 Personality traits questionnaire (Khromov, 2000; the internal consistency in the current study, *α* = 0.776). The latter was included because previous studies have revealed a relationship between personality traits and sugar consumption, as well as social conformity (Keller and Siegrist, 2015; Intiful et al., 2019; Parsad et al., 2019).

### 2.6. Procedure

Participants were told that the goal of the experiment was to study food preferences. They were asked to not eat anything for at least 3 h prior to the experiment (Hutcherson et al., 2012; Ntoumanis et al., 2022). This also limited the variability of their hunger level, which is a factor that has been shown to affect the amplitude of certain ERPs in response to food stimuli (Nijs et al., 2010b). Upon arrival at the laboratory, participants saw the real food products to be assured about the validity of the procedure. Then, they performed a practice session, where they had to bid on six of the products under the same conditions as in the subsequent experimental task. At the beginning of the experiment, participants performed a bidding task consisting of 90 trials (30 per condition). Next, they listened to the healthy eating call and afterward, they performed a second block of the bidding task. Figure 1B illustrates the experimental procedure. The stimulus presentation and response recording were controlled by PsychoPy (v2022.2.1; Peirce et al., 2019). On average, participants took approximately 1.5 h to complete the experiment, including the EEG setup.

### 2.7. Behavioral data analysis

The hypothesis that the healthy eating call would decrease the WTP for sugar-containing food, but would not change the WTP for sugar-free and non-edible products was specified prior to data collection, based on the results of Ntoumanis et al. (2022). To test this hypothesis, a one-way, repeated-measures ANOVA taking Condition (three levels: sugar-containing, sugar-free, non-edible) as a within-subjects factor and the *ΔWTP* (i.e., the WTP for each product in the second block subtracted by the WTP for the same product in the first block) as a dependent variable was conducted. A significant interaction was further assessed by post hoc tests. Specifically, given the normal distribution of the data (as assessed by a Shapiro-Wilk test, *p* > 0.05), we conducted pairwise paired-samples *t*-tests, and *p*-values were corrected for multiple comparisons using the Benjamini-Hochberg false discovery rate (FDR) correction (Benjamini and Hochberg, 1995). Adjusted *p*-values below 0.05 were considered statistically significant. Moreover, in order to investigate the efficacy of expert persuasion separately for each condition, we conducted one-sample *t*-tests to determine whether the delta of WTP for each condition was significantly different from 0 (two-tailed). The above analyses allowed us to test the hypothesis (H1).

To explore the relationship between consumer characteristics and the efficacy of expert persuasion, a series of correlation analyses was conducted. For this, we examined whether participants’ delta of WTP for each condition was significantly correlated with their questionnaire data using the Spearman’s correlation coefficient (due to the non-normal distribution of the questionnaire scores). This analysis was exploratory and no hypotheses were specified in advance.

### 2.8. EEG data recording and pre-processing

The EEG activity was recorded continuously with a BioSemi Active Two system at a sampling frequency of 500 Hz. Subjects were fitted with a standard, 64-electrode cap following the international 10–10 system, with linked mastoids as a reference electrode. To subsequently remove eye-movement artifacts, the vertical and horizontal electrooculogram (VEOG and HEOG) were also recorded with two auxiliary electrodes (one located ventrally to one eye and one located laterally to the other eye). In order to achieve a precise time alignment of all the stimuli presentations (including pictures and the healthy eating call), the stimulus presentation software sent triggers to a parallel port simultaneously with the presentation of stimuli. The timing of these triggers was set to be synchronized with screen refresh so that it captures the actual instead of the expected onsets. All offline signal processing and artifact correction was performed in MNE Python (v1.0.3; Gramfort et al., 2013).

For the ISC analysis, the data were preprocessed following the procedure described in Dmochowski et al. (2012) and Ntoumanis et al. (2023). First, data were re-referenced to average reference (Shtyrov et al., 2013). Next, the segment of the EEG/EOG signal corresponding to the duration of the healthy eating call was extracted based on the triggers sent by the stimulus presentation software to a parallel port at the onset and offset of the stimulus. We further excluded the first 15 and the last 5 s of this segment, as it is recommended in Nastase et al. (2019), to avoid including in the analysis changes in the signal driven by the onset and offset of the stimulus. Data were downsampled at 250 Hz, high-pass filtered at 0.5 Hz, and notch-filtered at 50 Hz and 100 Hz, in order to remove drift and power line noise, respectively. Afterward, noisy channels were detected by visual inspection and the samples of these channels were interpolated based on the signals of the good sensors around them (Ki et al., 2016; on average 4.5 channels in one recording). Eye-movement artifacts were removed by Independent Component Analysis (ICA) using the infomax algorithm (Bell and Sejnowski, 1995). Samples exceeding 3 SDs of the mean of their respective channel were replaced with 0, and so were the samples 40 ms around such outliers (i.e., before and after; Cohen and Parra, 2016; Ntoumanis et al., 2023). For the ERP analysis, we preprocessed the data in the same way except that a different band-pass filter was applied: 0.1 Hz and 40 Hz cut-off frequencies, following previous ERP studies (Bredikhin et al., 2022). Also, the outliers in the ERP signals were detected based on the interquartile interval (IQR) instead of the SD, as in Rappaport et al. (2019). In fact, epochs containing samples beyond the range [Q1−1.5×IQR, Q3+1.5×IQR], where Q1 and Q3 denote the 25th and the 75th percentiles, were rejected.

### 2.9. Intersubject correlation analysis

The ISC of EEG responses to the healthy eating call was estimated *via* a correlated components analysis (CorrCA; Dmochowski et al., 2012; Cohen and Parra, 2016). Briefly, this analysis finds components of the EEG data that are maximally correlated among subjects. Following previous studies, we estimated the ISC as the sum of the three most correlated components, in order to account for the overall neural synchronization regardless of the anatomical origin of each component (Cohen and Parra, 2016; Cohen et al., 2018; Ntoumanis et al., 2023). However, we also computed the forward model for each of the three strongest components to visualize the spatial distribution of the component activity (Cohen and Parra, 2016). The ISC was calculated in a leave-one-out approach, i.e., for each participant, there was one value denoting how correlated this participant’s brain activity was to the brain activity of all other participants during listening to the healthy eating call (Cohen and Parra, 2016; Ntoumanis et al., 2023). Taking into account the long duration of the narrative (7 min), we computed the ISC in sliding time windows of 10 s size and 8 s overlap (196 time windows in total). We selected this size based on a recent study which showed that the ISC can be most reliably measured on a time scale of 10 s (Madsen and Parra, 2022). The ISC analysis was performed in Matlab (release 2017b; MathWorks Inc, USA) using an adjusted version of the code shared by Cohen and Parra (2016)^1^. After calculating the leave-one-out ISC, we examined whether it was significantly correlated with the delta of WTP for each condition, using the Spearman’s correlation coefficient. This allowed us to test the hypothesis (H2).

### 2.10. Multivariate pattern analysis

An MVPA was conducted to investigate whether distributed patterns of EEG responses to the healthy eating call were predictive of the efficacy of expert persuasion (H3). Our analysis was similar to that conducted in Turner et al. (2017). First, we converted the delta of WTP for sugar-containing products to a binary variable: “highly-influenced” if the delta of WTP for sugar-containing food was less than the median score (22 participants) and “not highly-influenced”, otherwise (23 participants). A machine learning logistic regression classification model was then trained to predict, based on distributed patterns of EEG activity evoked by the healthy eating call, whether or not a participant was highly influenced by the narrative. This was done repeatedly in time windows of 1 s length, taking into account the long duration of the healthy eating call (7 min originally, 6 min 50 s after the removal of onset/offset; see EEG data recording and pre-processing). Specifically, the features/input of this classifier were the mean EEG signal of each channel within the corresponding time window. To avoid overfitting, a 5-fold cross-validation was performed, and the classification accuracy for each time window was calculated as the average percentage of correct guesses across all the cross-validation runs in the corresponding time window (Saarimäki et al., 2022).

Statistical testing was performed by comparing the classification accuracy to an empirical chance distribution instead of the theoretical chance level (in our case, 50%), because the latter is considered too lenient (Combrisson and Jerbi, 2015). In order to obtain an empirical chance distribution, we repeated the analysis described above 1,000 times, but each time with the labels (“highly-influenced”, “not highly-influenced”) randomly shuffled before classification (Turner et al., 2017). To correct for multiple comparisons, we used a cluster-based correction. Specifically, we clustered the windows with a statistically significant classification accuracy on the basis of temporal adjacency and finally, we took the largest of these clusters (Maris and Oostenveld, 2007). This approach allowed us to explore sustained information in the EEG signals that were predictive of the delta of WTP for sugar-containing food.

As a control analysis, we examined whether the spatiotemporal patterns of EEG responses to the healthy eating call could also predict the delta of WTP for non-edible and sugar-free products. To that end, we first classified individuals based on the median scores and we repeated the same machine learning analysis. Then, we compared the proportion of significant windows (i.e., windows where the classification accuracy was statistically significant) between conditions, using a standard hypothesis test of proportions, as in Dmochowski et al. (2012). We conducted this control analysis before correcting for multiple comparisons, because our cluster-based correction results by definition in only one significant cluster (Maris and Oostenveld, 2007), which makes further statistical testing difficult.

Finally, the feature weights were extracted for each time window with above chance classification accuracy and assigned to each channel. This provided us with a representation of the importance of each channel for the classification. Additionally, we repeated the same procedure outlined above with a different classifier (Support Vector Machine with linear kernel), but since it did not improve the classification results, it is not reported here. The MVPA analysis was performed in Python 3.10, using the Scikit learn package (Pedregosa et al., 2011).

### 2.11. ERP analysis

Based on the picture presentation at 0 ms (Figure 1A; “early evaluation phase”), grand average ERP epochs were selected from −100 to 1,000 ms. Baseline (from −S100 to 0 ms) correction was applied in each epoch. The P300 amplitude was calculated using the mean voltage of midline parietal electrodes (CPz, Pz, Oz) between 250 and 450 ms relative to the stimulus onset (Schubert et al., 2021). First, we examined whether the P300 amplitude in response to different product categories is modulated by the healthy eating call. To that end, we conducted a two-way repeated-measures ANOVA taking Condition (three levels: sugar-containing, non-edible, sugar-free) and Block (two levels: Block 1, Block 2) as within-subjects factors and the WTP as the dependent variable. This analysis allowed us to test our prespecified hypothesis (H4), but also to exploratorily investigate whether different product categories elicit different amplitudes of P300 in the first block (i.e., without any intervention). Next, we tested our prespecified hypothesis (H5) by calculating the Spearman’s correlation coefficient between the delta of WTP for sugar-containing products and the delta of P300 amplitude in response to sugar-containing products.

Moreover, we calculated the mean amplitude of one additional ERP component, the Late Positive Potential (LPP; Schubert et al., 2021), which was found to be elicited by viewing the products. This was calculated using the mean voltage of the same electrodes (CPz, Pz, Oz) between 450 and 750 ms relative to the stimulus onset, in line with previous studies (Schubert et al., 2021). Then, we conducted the same two-way repeated-measures ANOVA, as for the P300 amplitude, in order to examine whether the healthy eating call modulated the LPP in response to some products. This analysis was exploratory and no hypotheses were specified in advance.

## 3. Results

### 3.1. Descriptive statistics

The average bid was 47.39 MU (SD = 23.83 MU). Overall, 83.65% of all bids were higher than zero. One-sample Wilcoxon signed rank test showed that the median bid was significantly greater than zero (*W* = 22, 784, 625, *p* < 0.0001, effect size = 0.85), suggesting that most products were rewarding for the participants. The mean reaction time (RT) was 2.92 s (SD = 1.53), while participants failed to bid within the 5 s time limit only in 1.09% of the trials. As data from the first block showed, participants did not bid differently for sugar-containing, sugar-free and non-edible products (*F* = 0.033, *p* = 0.97, generalized eta-squared = 0.001).

The BMI of the participants ranged from 16.96 to 32.45, with the mean BMI being equal to 22.10. Four participants scored lower than 18.5 (underweight), eight participants scored above 25 (overweight) and 33 scored in between (normal). The majority of the participants had a complete (*N* = 19) or incomplete (*N* = 18) higher education, while the remaining participants (*n* = 8) had a complete secondary education. Finally, the scores in the personality questionnaires are summarized in Supplementary Table 1.

### 3.2. The healthy eating call decreased WTP for sugar-containing food

First, we investigated the influence of the healthy eating call on the WTP for sugar-containing, sugar-free and non-edible products. A repeated-measures ANOVA revealed a statistically significant effect of Condition on the delta of WTP (*F*_(2,88)_ = 6.876, *p* = 0.002, generalized eta-squared = 0.09). Subsequent paired *t*-tests showed that the delta of WTP for sugar-containing products was significantly lower than the delta of WTP for sugar-free (*t*_(44)_ = −3.24, *p* = 0.002, Cohen’s d = 0.48) and non-edible products (*t*_(44)_ = −2.19, *p* = 0.034, *d* = 0.33), while the delta of WTP for sugar-free food was not significantly different from the delta of WTP for non-edible products (*t*_(44)_ = 1.85, *p* = 0.070, *d* = 0.28). This demonstrates that the healthy eating call influenced participants’ WTP decisions for sugar-containing, but not for sugar-free food products, relative to the control condition of non-edible products.

**Figure 2:**
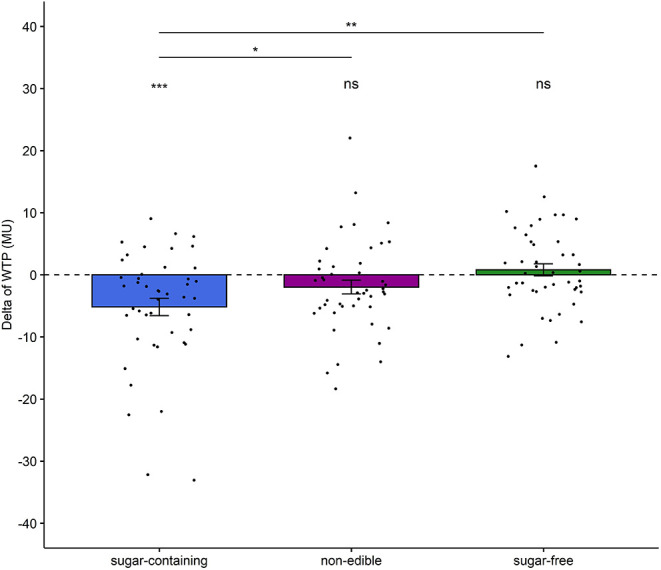
Changes in participants’ WTP for sugar-containing, sugar-free and non-edible products after listening to the healthy eating call. A dashed horizontal line at 0 indicates no change in WTP. Dots represent individual subjects. Statistically significant differences are denoted with asterisks (*p < 0.05, **p < 0.01, ***p < 0.001). The “ns” denotes that the delta of WTP for neither the sugar-free nor the non-edible products was significantly different from 0.

Apart from comparing the delta of WTP between conditions, we also examined whether this measure is significantly different from 0 for each condition separately. Independent one-sample *t*-tests showed that the delta of WTP for sugar-containing food products was significantly lower than 0 (*t*_(44)_ = −3.73, *p* = 0.0005, *d* = 0.56), unlike the delta of WTP for sugar-free and non-edible products (*t*_(44)_ = 0.82, *p* = 0.42, *d* = 0.12 and *t*_(44)_ = −1.78, *p* = 0.08, *d* = 0.27, respectively). Figure 2 illustrates these findings, which support our hypothesis (H1) that the healthy eating call would decrease individuals’ WTP for sugar-containing products.

### 3.3. The efficacy of expert persuasion was not moderated by participant demographic characteristics

An exploratory correlation analysis was conducted to examine whether the efficacy of expert persuasion is significantly moderated by participant’s demographic characteristics and personality traits. Due to the non-normal distribution of most variables, a Spearman’s correlation coefficient was used. A negative correlation was found between the delta of WTP for sugar-containing food and scores in the Conformity scale (*r* = −0.30, *p* = 0.049). This indicates that the healthy eating call was particularly influential on participants who are, in general, prone to social influence. Also, we found a significantly negative correlation between the delta of WTP for non-edible products and scores in the Neuroticism scale of the Big 5 Personality traits questionnaire (*r* = −0.51, *p* = 0.0004). No other significant correlation was found between the delta of WTP and any other participant characteristic (Supplementary Table 2). Moreover, we compared the delta of WTP for each condition between males and females, with the results, however, not reaching statistical significance (two-samples *t*-tests; *t*_(43)_ = 0.68, *p* = 0.51; *t*_(43)_ = 1.68, *p* = 0.104; *t*_(43)_ = 0.71, *p* = 0.48, for sugar-containing, non-edible and sugar-free, respectively).

### 3.4. ISC and efficacy of expert persuasion

**Figure 3:**
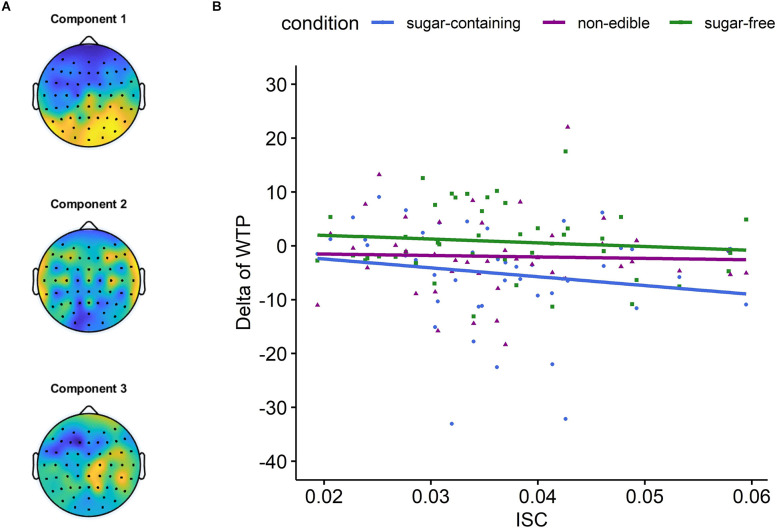
The results of the intersubject correlation (ISC) analysis. **(A)** Scalp projections of the three strongest correlated components. **(B)** The relationship between the ISC during listening to the healthy eating call and the delta of WTP for different product categories. For the category of sugar-containing products, the correlation was *r* = −0.29, with a one-tailed *p*-value = 0.027.

First, we estimated the three most correlated components using EEG data corresponding to the whole duration of the narrative (Figure 3A). These correlated components were found to be similar to previous studies (e.g., Dmochowski et al., 2012; Cohen and Parra, 2016). Specifically, the first component revealed a strong positivity at occipital sites, probably because all participants were looking at the same picture while listening to the narrative (see Figure 2). The second component revealed a symmetric positivity at temporal sites, consistent with the auditory processing of the narrative.

Then, we calculated the leave-one-out ISC during listening to the healthy eating call. Considering the long duration of the healthy eating call, the ISC was calculated in sliding time windows and then it was averaged across all the time windows for each participant. Next, we calculated the Spearman’s correlation coefficient between these ISC values and the delta of WTP for each product category, separately. The results showed that the ISC during listening to the healthy eating call was negatively correlated with the delta of WTP for sugar-containing food (*r* = −0.29, *p* = 0.027; Figure 3B), supporting our hypothesis (H2). Notably, this significant p-value was obtained from a one-tailed hypothesis test (i.e., *H*_0_: *r* < 0), since our hypothesis (H2) was directional. In addition, the correlation between the ISC and the delta of WTP for the other two product categories was not found statistically significant (*r* = −0.03 and *p* = 0.87 for sugar-free, *r* = −0.10 and *p* = 0.50 for non-edible).

### 3.5. MVPA

A machine learning classification analysis was conducted to investigate whether spatiotemporal patterns of the EEG responses to the healthy eating call could predict the efficacy of expert persuasion (that is, the delta of WTP for sugar-containing food). To that end, we first labeled half of the participants as “highly-influenced” and the other half, “not highly-influenced” by the healthy eating call, based on the median delta of WTP for sugar-containing products. Then, we conducted the MVPA in 1-s time windows.

The classification accuracy was statistically significant in 22.25% of the time windows (87 significant windows out of 400). After applying a cluster-based correction for multiple comparisons, there were two clusters whose classification accuracy remained statistically significant: one between the 165th and the 168th second of the healthy eating call and one between the 200th and the 203th. In other words, whether or not a participant was highly influenced by the healthy eating call could be best predicted by their EEG signal during these two periods. Specifically, in the first cluster (i.e., 165–168 s) the average classification accuracy was 65%, while the average accuracy of the empirical chance distribution was 49.5%. In the second cluster (i.e., 200–203 s) the average accuracy was 69%, while the average accuracy of the empirical chance distribution was 49.9%.

To better understand what was in the healthy eating call during these periods that evoked distinct neural responses between the participants who were highly influenced by it and those who were not, we extracted the corresponding text from the narrative. At the 160th second of the narrative, the following phrase was said: “Your skin will look younger. A study in the American Journal of Clinical Nutrition suggests that giving up sugar may result in your acne disappearing”, which finished at the 172th second. At the 198th second of the narrative, the following phrase was said: “Your blood pressure will also decrease, which means that your heart and your vessels will have less work to do”, which finished at the 204th second.

**Figure 4:**
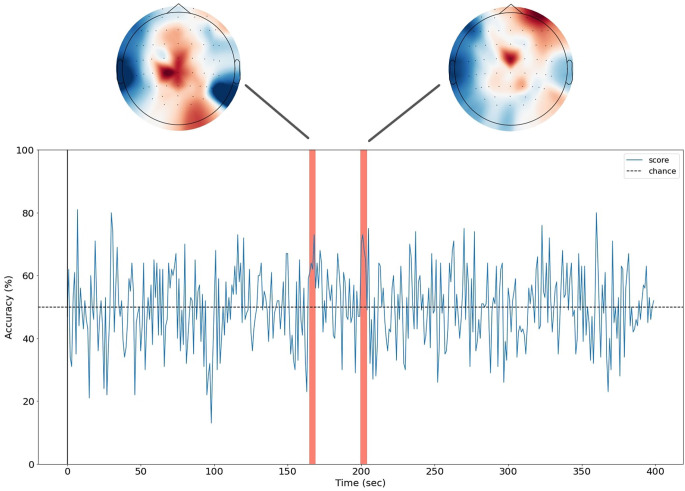
Spatio-temporal decoding of the delta of WTP for sugar-containing food. A machine learning logistic regression was used to predict the delta of WTP for sugar-containing products (binary variable) based on distributed patterns of EEG responses to the healthy eating call. The blue line denotes the classification accuracy for each time window. A horizontal dashed line has been added at 50% as a reference, although statistical testing was performed based on permutations. The red bars indicate the clusters where the classification accuracy was statistically significant after a cluster-based correction for multiple comparisons. The feature weights of the classifiers in the significant cluster windows have been added at the top of the plot. These are the standardized activation patterns.

Finally, we extracted the feature weights of the machine learning algorithm for the two significant clusters of time windows. Although these maps cannot determine the sources of predictive information (Haufe et al., 2014), they provide a representation of the importance of each channel for the classification (Figure 4). This revealed that electrodes at the temporal sites were the main contributors to the significant classification accuracy that was observed between the 165 and 168 seconds of the healthy eating call. For the second significant cluster, it appears that the signal of frontal electrodes provided sufficient information to the classifier.

As a control analysis, we repeated exactly the same MVPA for non-edible and sugar-free products. That is, we examined whether the EEG responses to the healthy eating call could also predict the delta of WTP for these two product categories. The classification accuracy was significant only in 27 time windows for non-edible products and in 16 time windows for sugar-free products. We employed a standard hypothesis test of proportions to test whether the observed ratios are drawn from disparate distributions, as in Dmochowski et al. (2012). This showed that the proportion of significant windows was significantly higher in the sugar-containing condition than in the other two conditions (both *p*-values < 0.0001; Supplementary Figure 1).

### 3.6. The healthy eating call did not modulate the P300 and LPP amplitudes in response to any of the products

First, we conducted a series of ANOVAs to investigate whether the healthy eating call modulated the neural responses to sugar-containing, sugar-free and non-edible products, irrespective of WTP decisions. In these analyses, the dependent variable was the amplitude of the ERP component of interest (P300 or LPP) and the within-subjects factors were always the Condition (three levels: sugar-containing, sugar-free, non-edible) and the Block (two levels: Block 1, Block 2).

There was a significant main effect of Condition on the P300 amplitude [*F*_(2,88)_ = 11.028, *p* < 0.0001, generalized eta-squared (ges) = 0.028] and a significant main effect of Block (*F*_(1,44)_ = 9.59, *p* = 0.003, ges = 0.012). However, the interaction between Condition and Block was not statistically significant (*F*_(2,88)_ = 0.34, *p* = 0.71, ges = 0.001). To better understand the significant main effects of Condition and Block, we conducted pairwise one-sample *t*-tests. These revealed that regardless of the Block, the P300 amplitude was weaker (less positive-going) for non-edible products compared to sugar-containing (*t*_(89)_ = 3.60, *p* = 0.001, *d* = 0.38) and sugar-free products (*t*_(89)_ = 4.96, *p* < 0.0001, *d* = 0.52), but there was no significant difference between the latter two conditions (*t*_(89)_ = 1.33, *p* = 0.19, *d* = 0.14). Also, regardless of the Condition, the P300 amplitude was stronger (more positive-going) in the second block of the bidding task compared to the second (*t*_(134)_ = 3.59, *p* < 0.001, *d* = 0.32). Finally, contrary to our hypothesis (H4), the P300 amplitude in response to sugar-containing food was significantly stronger (more positive-going) in the second block of the bidding task compared to the first (*t*_(44)_ = 2.65, *p* = 0.011).

**Figure 5:**
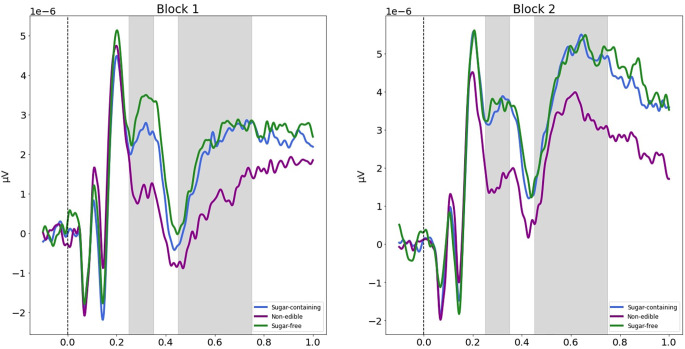
P300 and LPP components shaded in gray in two consecutive time windows (earlier and later, correspondingly) in response to each product category, in the first (left) and the second (right) block of the bidding task. In both blocks, the mean P300 amplitude and the mean Late Positive Potential (LPP) amplitude were found to be significantly lower for non-edible products compared to sugar-containing and sugar-free products. The gray areas indicate the time window in which the P300 and the LPP were measured, based on previous literature.

Similar results were found in terms of the LPP amplitude. Specifically, there was a significant main effect of Condition on the LPP amplitude (*F*_(2,88)_ = 7.11, *p* = 0.001, generalized eta-squared (ges) = 0.021) and a significant main effect of Block (*F*_(1,44)_ = 24.55, *p* < 0.0001, ges = 0.07). However, the interaction between Condition and Block was not statistically significant (*F*_(2,88)_ = 0.04, *p* = 0.96, ges < 0.0001). To better understand the significant main effects of Condition and Block, we conducted pairwise one-sample *t*-tests. These revealed that regardless of the Block, the LPP amplitude was weaker (less positive-going) for non-edible products compared to sugar-containing (*t* = 3.42, *p* = 0.001) and sugar-free products (*t* = 3.54, *p* = 0.001), but there was no significant difference between the latter two conditions (*t* = 0.27, *p* = 0.79). Also, regardless of the Condition, the LPP amplitude was stronger (more positive-going) in the second block of the bidding task compared to the second (*t* = 6.74, *p* < 0.0001). Figure 5 illustrates the differences between the P300 and LPP amplitudes between conditions and blocks.

Finally, we tested our hypothesis (H5) by calculating the Spearman’s correlation coefficient between the delta of the P300 amplitude and the delta of WTP for each product category. However, no significant correlation was found: *r* = 0.10, *p* = 0.51 for sugar-containing, *r* = 0.1, *p* = 0.520 for non-edible, and *r* = −0.045, *p* = 0.77 for sugar-free products.

## 4. Discussion

This study presents a first attempt to link, on the one hand, the behavioral effect of healthy eating by an expert on the WTP for sugar-containing food and, on the other hand, the neural responses to this healthy eating call with the use of EEG. At the behavioral level, the results illustrated a successful persuasion by the health expert. This behavioral effect was associated with two neurophysiological indices, namely, (i) the group-level ISC of EEG responses to the healthy eating call and (ii) the subject-level spatiotemporal patterns of EEG responses to the healthy eating call. Below, we further consider these findings in more detail.

The analysis of the behavioral data showed that the healthy eating call significantly decreased participants’ WTP for sugar-containing food. This result is consistent with our hypothesis (H1) and replicates the recent findings of Ntoumanis et al. (2022). It also supports earlier research showing that healthy eating calls can, in general, be effective at reducing unhealthy eating (Mollen et al., 2013; van Kleef et al., 2015). In fact, our healthy eating call was a first-person narrative by an expert, which is a type of intervention that has been found to be effective in children populations, too (Binder et al., 2020). We speculate that an advantage of using first-person narratives, over such types of nudging as labeling, is their potential to change consumers’ underlying perception of unhealthy food instead of targeting individual decisions. Narratives can shape social norms, injunctive or descriptive, which can support their effectiveness (Robinson et al., 2013; Higgs, 2015; Higgs and Thomas, 2016).

In addition, the healthy eating call did not increase the WTP for sugar-free food (relative to the control condition of non-edible products). That the healthy eating call was more effective at reducing the WTP for sugar-containing food than at increasing the WTP for sugar-free food is supported by previous studies showing that nudge interventions are, in general, more efficient at reducing unhealthy eating than increasing healthy eating (Zlatevska et al., 2014; Cadario and Chandon, 2020), as well as by the concept of negativity bias (Baumeister et al., 2001).

It is worth mentioning that the current study included a control condition (non-edible products) and not a control group of subjects. However, our previous work showed that a control group of subjects, who listened to a control message unrelated to food, did not change their WTP for sugar-containing or sugar-free food (Ntoumanis et al., 2022).

Interestingly, we found that the delta of WTP for sugar-containing food was negatively correlated with the Conformity scale scores. The Conformity scale measures the reliance on others for decision-making, in a variety of social contexts (Mehrabian and Stefl, 1995). Thus, healthy eating calls may be particularly effective on consumers who are, in general, susceptible to social conformity. Our study is not the first to show that conformity moderates consumers’ decision-making. For instance, Martinelli and De Canio (2021) illustrate the moderating role of conformity in inducing non-vegan consumers to buy vegan food. Therefore, we encourage future studies to sample personality-related information from the participants, to account for the intersubject variability in consumer behavior (for a review, see Kassarjian, 1971).

Furthermore, we found a significant one-tailed correlation between the ISC during listening to the healthy eating call and the efficacy of expert persuasion. In fact, participants, whose neural responses to the expert’s narrative correlated more strongly with others, demonstrated superior compliance with the healthy eating call. Although this result has to be considered with caution due to the statistical insignificance of the two-tailed correlation (*p* = 0.054), it is consistent with our hypothesis (H2). ISC of EEG has previously been found to be positively correlated with subsequent memorization of audio narratives (Cohen and Parra, 2016). In our study, participants who exhibited high ISC during listening to the healthy eating call might have memorized more information contained in it, which might have increased their compliance with the expert. Overall, neural synchrony is a promising tool in neuroforecasting for movie and music popularity (Christoforou et al., 2017; Leeuwis et al., 2021), as well as for television ads (Dmochowski et al., 2014). Here, we show that neural synchrony may, in addition, be a promising tool in neuroforecasting of healthy eating advertisements.

Moreover, distributed patterns of brain activity during listening to the healthy eating call were found to contain predictive information about whether or not a participant was highly influenced by the healthy eating call. Although not all, several arguments in the narrative elicited divergent neural responses to participants who were highly influenced by them. Training a machine learning classifier with EEG data corresponding to those arguments significantly predicted whether or not a participant was highly influenced by the healthy eating call. Previous works have successfully predicted consumer decision-making based on EEG responses to non-naturalistic stimuli (Bode et al., 2014; Turner et al., 2017). Our proof-of-concept MVPA shows that it is also possible to predict consumer decision-making based on EEG responses to naturalistic stimuli, supporting our hypothesis (H3).

It is interesting to speculate what are the cognitive mechanisms that highly-influenced participants employed while listening to those arguments, which resulted in their neural activity being discernible. Information encoding is a possible such mechanism. That is, while listening to the health risks of sugar, highly-influenced participants might have encoded this information differently from not highly-influenced participants. Another interpretation could be that the information contained in the narrative activated some other related cognitive processes (e.g., knowledge retrieval) only in highly-influenced participants. Determining which of these two possible interpretations is true is a well-known challenge in MVPA (Weaverdyck et al., 2020). Despite the low spatial resolution of EEG, we can speculate which interpretation is the most likely, based on the scalp representation of the feature weights of the classifier (Figure 4). The electrodes that contributed the most in the classification process were located in temporal and frontal sites, supporting the information encoding interpretation. In fact, previous studies have reported that these brain areas show increased activity when one is feeling persuaded (Falk et al., 2010).

Furthermore, contrary to our hypothesis (H4), the healthy eating call did not decrease the P300 amplitude in response to sugar-containing food. In fact, the P300 elicited by sugar-containing products was even more positive going in the second vs. the first block of the bidding task. Our hypothesis was based on previous studies showing that the more we want a product, the stronger the P300 we exhibit when viewing it. However, the P300 can be elicited by stimuli of negative valence, as well (Conroy and Polich, 2007; Schienle et al., 2008). In our study, we speculate that pictures of sugar-containing food might induce higher levels of fear after the healthy eating call compared to the first session of the bidding task. This is supported by Schienle et al. (2008) who showed that fearful pictures elicit stringer P300 compared to neutral pictures. Consequently, our hypothesis (H5) was not supported, either.

Moreover, although not related to our main hypotheses, we found that both before and after the healthy eating call, non-edible products elicited weaker (less positive-going) P300 and LPP responses compared to edible products (sugar-containing or sugar-free). Earlier research using EEG to derive attention-related neural responses to food vs. non-food has revealed similar results (Nijs et al., 2008, 2009, Nijs et al., 2010a,b). This phenomenon has been explained on an evolutionary basis, that is, selective attention to food is an important characteristic of humans and animals (Nijs et al., 2010b).

Our study design has several advantages. First, the within-subjects design allowed us to measure the effect of the healthy eating call while minimizing the noise of intersubject variability. Second, participants made real choices—they were told that they would receive a product at the end of the experiment, which is considered to encourage sincere WTP ratings (Plassmann et al., 2007; Schubert et al., 2021). Third, the incorporation of EEG provided us with the opportunity to investigate potential neural signatures of the healthy eating call’s behavioral effect.

Also, our study has several limitations. For example, as in Ntoumanis et al. (2022), we used a sugar-containing label to ensure a clear discrimination between the conditions of our experiment. However, labels highlighting the content of sugar in products are not common in the country where the experiment was conducted. Thus, even if marketing companies, inspired by our healthy eating call, manage to successfully incorporate a similar narrative in advertisements to influence consumers against sugar, it would be more difficult for the consumers, than it was for our participants, to later spot and avoid sugar-containing food. In addition, unlike in the real market, the products were presented in our experiment without packaging. An attractive packaging, which is an important factor underlying food valuation (Motoki and Suzuki, 2020), may override the effects of a healthy eating call. Another limitation is the low spatial resolution of EEG, which does not allow for accurate localization of the effects. For instance, we found a relationship between the whole-brain ISC and the efficacy of expert persuasion, but using the ISC in specific brain regions might improve the significance of this result. To address this, future fMRI studies could conduct an intersubject representational similarity analysis (IS-RSA; Finn et al., 2020). Finally, we did not ask the participants to rate how impactful each argument presented in the healthy eating call is to them. This additional data would allow us to assess whether the MVPA classification accuracy co-varies with the importance of the arguments.

Taken together, our work contributes to our understanding of how healthy eating calls can reduce the WTP for sugar-containing food, which is important considering that sugar is the key cause of the growing obesity rates (Yu et al., 2022). By using EEG, we elucidated the neural mechanisms by which the brain responds to persuasive messages by experts. From a broader perspective, our results demonstrate that EEG is a powerful tool that can be used to predict the efficacy of health-related advertisements before they are released to the public.

## Data availability statement

The raw data supporting the conclusions of this article will be made available by the authors, without undue reservation.

## Ethics statement

The studies involving human participants were reviewed and approved by the Institutional Review Board, Higher School of Economics, Moscow, Russia. The patients/participants provided their written informed consent to participate in this study.

## Author contributions

IN: conceptualization, methodology, software, validation, formal analysis, investigation, resources, data curation, writing—original draft, writing—review and editing, visualization, and project administration. AD and JS: investigation. KP: conceptualization, writing—review and editing. VKo and AS: writing—review and editing. IJ: writing—review and editing, funding acquisition. VKl: conceptualization, writing—review and editing, funding acquisition. All authors contributed to the article and approved the submitted version.

## Footnotes

^1^ https://www.parralab.org/isc/
