# Supplementary material for: Neural mechanisms of expert persuasion on willingness to pay for sugar
Source: Front Behav Neurosci. 2023 Mar 13;17:1147140. doi: 10.3389/fnbeh.2023.1147140 (PMC10040640; doi:10.3389/fnbeh.2023.1147140)
Supplement: Supplementary file 1 [file Data_Sheet_1.pdf]

Supplementary Material

Neural mechanisms of expert persuasion on willingness to pay for sugar

Ioannis Ntoumanis[*,1], Alina Davidova[1], Julia Sheronova[1], Ksenia Panidi[1], Vladimir Kosonogov[1], Anna Shestakova[1], Iiro P. Jääskeläinen[1,2], Vasily Klucharev[1]

* Correspondence: Ioannis Ntoumanis, iannisntoumanis@yahoo.com

Supplementary Figures

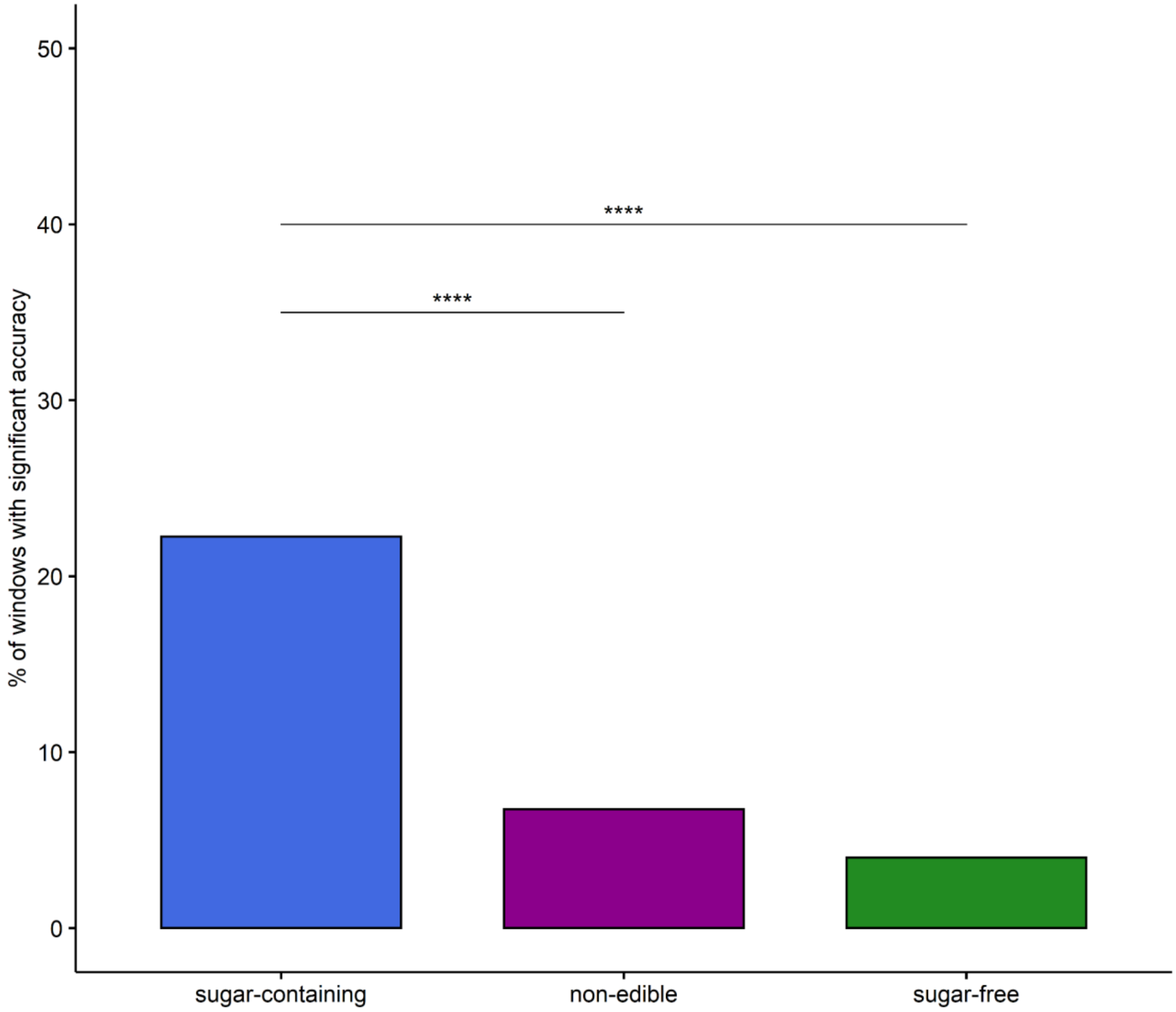

Supplementary Figure 1. Percentage of time windows where the classification accuracy of the MVPA was statistically significant. The proportions for the conditions sugar-containing, non-edible and sugar-free were 22.25%, 6.75% and 4.00%, respectively.

Supplementary Tables

Supplementary Table 1

Scores in the personality questionnaires.

	Mean	Actual range	Possible range
Conformity	4.46	2.18 - 7.36	1 - 9
Consumer susceptibility to interpersonal influence scale	3.45	1.08 - 5.42	1 - 7
Extraversion	2.93	1.47 - 4.20	1 - 5
Openness	3.81	2.73 - 4.53	1 - 5
Agreeableness	3.47	2.20 - 4.33	1 - 5
Neuroticism	3.23	1.13 - 4.73	1 - 5
Conscientiousness	3.23	1.27 - 4.47	1 - 5

Supplementary Table 2

Relationship between participant characteristics and change in willingness to pay for sugar-containing products after listening to the healthy eating call. The numbers in the cell denote the Spearman's correlation coefficient. *p<.05, ***p<.001.

	sugar-containing	non-edible	sugar-free
Age	.02	.10	.08
BMI	-.21	-.24	.11
Social conformity	-.30*	-.16	-.10
Consumer susceptibility to interpersonal influence scale	-.04	.09	.07
Openness	-.13	.08	.04
Agreeableness	.15	.21	-.27
Conscientiousness	-.26	.12	-.06
Neuroticism	-.27	-.51***	.016
Extraversion	.13	.25	-.05

Note: We also examined whether the change in WTP for each product category was different between participants with different levels of education. Three ANOVA were conducted (one for each condition) with Education as a between-subjects factor, but none of them showed a significant relationship between education and change in WTP (all ps > .05).
